# Artificial light at night causes conflicting behavioural and morphological defence responses in a marine isopod

**DOI:** 10.1098/rspb.2023.0725

**Published:** 2023-06-14

**Authors:** Kathryn Bullough, Kevin J. Gaston, Jolyon Troscianko

**Affiliations:** ^1^ Centre for Ecology and Conservation, University of Exeter, Penryn TR10 9FE, UK; ^2^ Environment and Sustainability Institute, University of Exeter, Penryn TR10 9FE, UK

**Keywords:** artificial light at night, camouflage, colour-change, lightstructure, *Ligia oceanica*, visual ecology

## Abstract

Encroachment of artificial light at night (ALAN) into natural habitats is increasingly recognized as a major source of anthropogenic disturbance. Research focussed on variation in the intensity and spectrum of ALAN emissions has established physiological, behavioural and population-level effects across plants and animals. However, little attention has been paid to the structural aspect of this light, nor how combined morphological and behavioural anti-predator adaptations are affected. We investigated how lighting structure, background reflectance and the three-dimensional properties of the environment combined to affect anti-predator defences in the marine isopod *Ligia oceanica.* Experimental trials monitored behavioural responses including movement and background choice, and also colour change, a widespread morphological anti-predator mechanism little considered in relation to ALAN exposure. We found that behavioural responses of isopods to ALAN were consistent with classic risk-aversion strategies, being particularly exaggerated under diffuse lighting. However, this behaviour was disconnected from optimal morphological strategies, as diffuse light caused isopods to become lighter coloured while seeking out darker backgrounds. Our work highlights the potential for the structure of natural and artificial light to play a key role in behavioural and morphological processes likely to affect anti-predator adaptations, survival, and ultimately wider ecological effects.

## 1. Introduction

With 80% of the global human population living under light-polluted skies [1], and sky brightness doubling every 8 years [2], artificial light at night (ALAN) is now of widespread concern in a range of habitats and many conservation priority areas [3,4]. This is particularly true of coastal habitats, with 22% of shorelines worldwide suffering light pollution [5] and 75% of global megacities in coastal regions [6]. ALAN is associated with detrimental effects in a wide range of biological processes, from gene expression to ecosystem functioning [7–12], including intertidal community composition [13,14]. Vision is a key sensory modality for many nocturnal predator and prey species, and elevated nocturnal light intensity—whether from natural or artificial sources—drives their activity patterns and alters their behaviour (e.g. [12,15–18]). ALAN will therefore affect direct mortality (due to light benefiting visually guided predators), or cause behavioural shifts associated with perceived predation risk (hiding to avoid predators, or increasing activity if the light makes approaching predators more visible). Indeed, responses to ALAN can tend to be more intense in areas where animals are exposed to higher levels of predation, linking predation activity to ALAN exposure [19].

Motion detection is one of the key visual signals that predators use to detect their prey [20,21] and could partially explain the above link between light and activity. Movement strategies such as intermittent motion and freeze responses are widespread in animals and are thought to interfere with a predator's ability to locate prey following a short burst of movement [22–24]. Animals can also regulate their movement strategies based on visual motion in their surroundings, such as the stop-start swaying motion of stick insects that is modulated by background foliage swaying [25]. Prey species might therefore be expected to modulate their movement strategy based on perceived predation risk, which for many species will be highly dependent on nocturnal light intensity.

For motionless animals, camouflage will often be their primary line of defence [26] and they can optimize this through two main mechanisms, morphological and behavioural. Adaptations that affect appearance/morphology enhance camouflage strategies such as background matching and edge disruption [27], or interfere with predator learning [28,29]. Many species, including butterfly larvae, crabs and fish, are also able actively to alter their appearance to match their surroundings [30–32]. This mechanism has rarely been explored in the context of ALAN, although Moorish geckos (*Tarentola mauritanica*) can change colour to background-match when ALAN is present, but not under natural dark conditions [33]. Similarly, cuttlefish are able to background-match under very low light levels, but not below 0.0001 lux [34]. Behavioural optimization of camouflage is achieved by selecting microhabitats that enhance the above camouflage mechanisms. This has been demonstrated in diurnal species such as crickets, lizards, ground-nesting birds and many other taxa [35–38], but remains poorly understood in nocturnal settings. A failure of either defence mechanism (inappropriate colour change or background selection) is likely to increase predation risk. For example, a treefrog that occurs in either brown or green morphs was shown to select colour-matching backgrounds, and experiments showed that frogs on colour-mismatched backgrounds were far more likely to suffer predation by garter snakes [39]. In a nocturnal context, seeking out dark refuges is a related strategy that many animals exhibit when exposed to bright ALAN, likely driven by negative phototaxis [40,41]. When prey hide in shadows that are sufficiently dark, predator vision won't be able to detect them, making the strategy more akin to occlusion than camouflage. However, if the receiver's vision is sufficiently sensitive, or the shadows insufficiently dark, camouflage will be essential for survival.

To date, research into the biological impacts of ALAN typically characterizes lighting based on its intensity and/or spectral emissions [12]. However, another key property is the directional structure of the light. This can vary from isotropic (diffuse)—with roughly equal light intensities from all directions that casts weak shadows—to highly directional (direct), such as a single-point light source that creates clear, dark shadows. Daytime light structure is known to have dramatic effects on natural visual scene properties, influencing fundamental anti-predator adaptations such as countershading [22,42,43], body posture [44], predator attack behaviour and prey vigilance behaviour [45–47]. The nocturnal light environment is even more spatio-temporally complex than that of the daytime; with lunar position, lunar phase, starlight and atmospheric conditions altering its intensity and directionality. ALAN can easily overwhelm these natural sources and brings with it substantial structural complexity as it can vary from direct (e.g. near a single streetlamp) to highly diffuse. Diffuse ALAN can result from multiple distributed sources, or by the reflection and scattering of light in the atmosphere creating skyglow [48,49]. Thus, while one of the most striking differences between natural light at night and ALAN is the structure of the light environment, this has rarely been considered in research on the impact of ALAN on animal behaviour and morphology [12]. To date there has only been one study that has explicitly compared the effects of direct and diffuse light, carried out around 80 years ago on the copepod *Acartia tonsa* [50]. This investigation found that diffuse lighting in the water column disrupted the copepod's vertical migrations compared to direct lighting, highlighting the importance of using experimental light structures that reflect those of real-world conditions [51]. Light structure alters background appearance and would therefore be expected to influence camouflage efficacy and strategy. For example, the shadows created by three-dimensional (3D) habitat features under direct light will increase the visual complexity of the scene, making the visual task more difficult for predators [52]. Conversely, diffuse light should aid the visual task of camouflage breaking and motion detection*.* However, the effect of nocturnal light structure on anti-predator defences remains entirely unexplored.

Perceived predation risk in prey is hypothesized to be modulated by interactions between light intensity, light structure, 3D microhabitat structure and background coloration. We investigated these effects in the sea-slater (*Ligia oceanica*), an abundant nocturnal species of isopod found on rocky shores across Europe. A number of factors make this species well suited for this study. (i) Closely related isopods have been found to undergo retinal cell structural changes at night to improve sensitivity to green wavelengths [53], implying their night vision regulates behaviour, and making them sensitive to artificial lighting [54,55]. (ii) They are able to change colour to match their surroundings and will select microhabitats that complement their own coloration [56,57], demonstrating both morphological and behavioural anti-predator defence mechanisms [58]. (iii) They are highly active prey species occupying exposed habitats and rely on camouflage as their primary defence mechanism. Gulls and other shorebirds are visually guided predators known to increase activity under high-nocturnal light levels [17,59,60] and are likely to target large isopods such as sea-slaters [61,62] (J.T. 2020–2023, unpublished data from local fieldwork). This means perceived predation risk in sea-slaters is likely linked to light levels, so should affect their movement behaviour.

**Figure 1.**
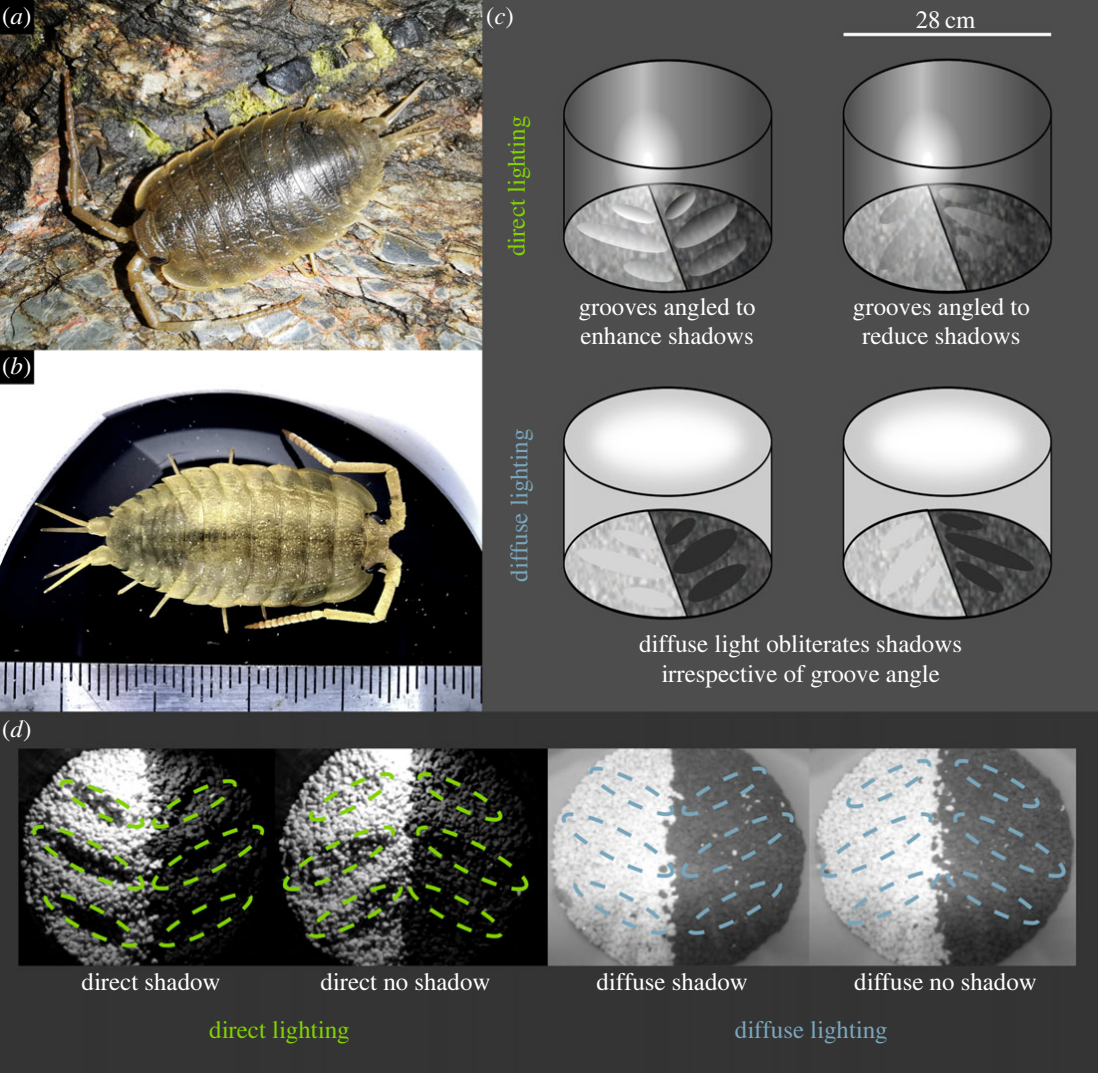
Figure showing examples of sea-slaters and the four experimental treatments used to determine their responses to different types of artificial lighting and shadows. Each treatment also offered a choice of a white or black gravel background. (*a*) A sea-slater foraging at night. (*b*) A photograph with scale-bar (1 mm notches). (*c*) Illustration of the set-up; lighting was either direct or diffuse, and the grooves in the gravel were either angled to enhance or reduce shadows: ‘shadow’ treatments were created by forming three grooves in the gravel on each half of the chamber, either perpendicular to the direction of the light to create shadows that were larger than the body size of the sea-slater being trialled, or parallel to the direction of the light to reduce larger shadows. (*d*) Still frames from the four treatments. However, note that diffuse light obliterated any shadows, so only the ‘direct shadow’ treatment created strong shadows. Dashed-line overlays show the groove angles.

We tested the morphological and behavioural anti-predator responses of sea-slaters using experimental chambers that had either direct or diffuse lighting (with intensities and spectra matched to typical habitat levels near coastal streetlights). Each chamber had 3D structures (grooves) in the gravel substrate aligned so that these either cast or eliminated shadows. Each chamber also offered a choice of pale (white) or dark (black) gravel substrate (figure 1). The wild-caught sea-slaters in our study were always a closer match to the black gravel than the white. Taken together with the concepts above, we would therefore predict that sea-slaters should spend more time on black gravel, change colour to match the black gravel, and they should exhibit the most risk-averse behaviour under diffuse light and on white backgrounds because these leave them most exposed to predator vision (i.e. sea-slaters should adopt fast, intermittent movement, and more pronounced background choice and colour change).

## 2. Methods

### (a) Data collection

Experiments were conducted at night over four months (September–December 2021) at Swanpool Beach, Falmouth, UK. Each trial consisted of a single, unique sea-slater being placed into an experimental chamber with an infrared video camera recording its movement behaviour for 15 min. Experimental chambers were constructed from circular buckets 28 cm in diameter that blocked all external illumination. The diffuse-lighting chamber had white walls and a diffuser introducing light uniformly from above. The direct-lighting chamber had black walls and a single-point light source *ca* 10 cm from the gravel substrate. Both chambers used the same model of white LED (Lumileds luxeon C 5700 K, RS, see electronic supplementary material, figure S1 for spectral emissions). The light intensity in both chambers was calibrated using driver circuitry so that a Spectralon 99% standard in the centre reflected 1 cd m^−2^ of light (an intensity equivalent to being approximately 10–30 m away from a typical streetlight), measured with a JETI specbos 1211-2. In both chambers, the floor was divided in half so that black gravel was present on the right and white gravel was present on the left. Three grooves were made in the gravel on each half of each chamber, either perpendicular to the direction of light to enhance large shadows, or parallel to the direction of light to reduce large shadows, as determined from the ‘direct-lighting’ chamber. Thus, chambers created four experimental treatments: ‘direct shadow’, ‘direct no shadow’, ‘diffuse shadow’ and ‘diffuse no shadow’, each with the choice of a black or white background (figure 1). Given the diffuse light treatment obliterated shadows, the ‘diffuse no shadow’ treatment did not contain shadows, but we have retained this label to act as a control for groove angle relative to the ‘direct shadow’ treatment. The ‘direct no shadow’ treatment also contains small shadows created by the gravel itself, thus our experimental design focuses on shadows larger than the sea-slaters themselves.

Sea-slaters were collected from the rocks south of Swanpool beach using a red LED headtorch to minimize disruption to their vision [53] and placed in a black-out box with no lighting for at least 15 min to acclimatize prior to running an experimental trial. Previous work (J.T. 2020–2023, unpublished data) suggests that this time is sufficient to elicit colour change, as do the experiments by Willmer *et al*. [57]. Individuals were photographed immediately before and after each trial for calculating start colour and colour change. This required a brief LED flash exposure that was kept short and consistent to limit photobleaching. Photographs were taken with a CUBOT Quest Lite phone, and a Zenith sintered PTFE diffuse 7% reflectance standard was used to calibrate images. Following a trial, each sea-slater was marked using a non-toxic black marker pen to avoid recapture and released at the capture site.

### (b) Data processing

Photographs and video footage were processed using ImageJ version 1.53n and the micaToolbox [63]. Photographs were calibrated against the grey standard and converted to blue tit relative double cone catch quanta (representing ecologically relevant predator vision). Regions of interest (ROIs) were then drawn over the main body of the sea-slater, and measured. Videos were processed using custom-written code in ImageJ. First, ROIs were drawn over the white background, the black background, and in the direct-lighting treatments the grooves on the white background, and the grooves on the black background (these grooves are not visible under diffuse light). Sea-slater movement in videos was tracked by manually clicking their location in 2 s intervals over the entire 15 min trial. For each trial, the code calculated the proportion of time spent in each ROI, speed and the s.d. of speed (used as a measure of intermittent motion).

### (c) Data analysis

Generalized linear models were used to analyse colour change and overall background choice behaviour, using Gaussian or quasibinomial error structures as appropriate. Zone-specific behavioural responses were analysed with generalized linear mixed effects models to account for repeat measures of individuals. Models were fitted with full interactions between experimental treatment levels, and these models were simplified using *χ*^2^, *F*-tests or AIC to eliminate higher level interactions. All analyses were carried out using RStudio version 3.6.3 (including the packages ‘lme4’ version 27.1, ‘ggplot2’ version 3.3.5, ‘tab’ version 5.1.1, ‘sjPlot’ version 2.8.10 and ‘ggpubr’ version 0.6.0). Raw data and R script are included as electronic supplementary material.

## 3. Results

A total of 155 trials—each using a unique sea-slater—were recorded and analysed (38 for direct shadow, 40 for direct no shadow, 37 for diffuse shadow and 40 for diffuse no shadow).

### (a) Background choice behaviour

**Figure 2.**
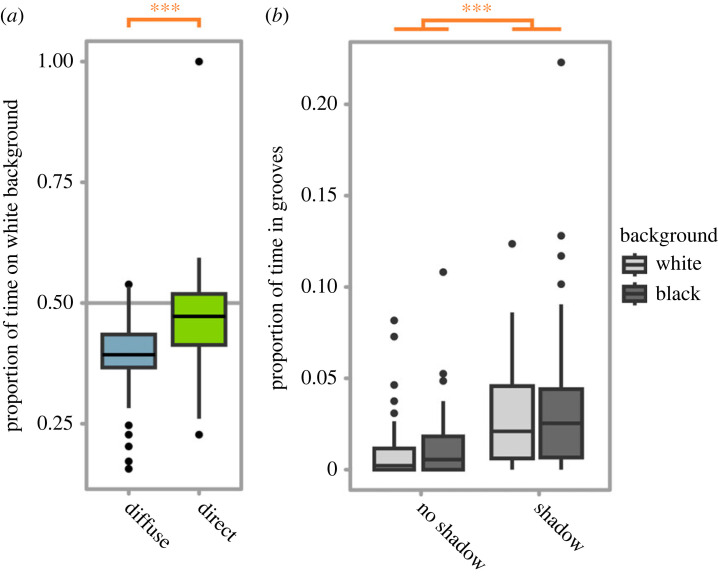
(*a*) The proportion of time sea-slaters spent on a white background under either diffuse or direct lighting. Under both types of lighting sea-slaters spent significantly less time on the white background, and this effect was much stronger under diffuse lighting. (*b*) The proportion of time sea-slaters spent in the grooves located on white and black backgrounds under the presence or absence of shadows. For both backgrounds, sea-slaters spent significantly more time in the grooves when shadows were present. Boxes represent the median and interquartile range, and whiskers represent the range of the dataset. *** indicates *p* < 0.001.

Sea-slaters spent more time on the black background under both direct and diffuse light (mean of 53.11% [±9.88] and 61.08% [±6.88] of time respectively), and this preference was significantly stronger under diffuse light (GLM, *t* = 3.74, *p* < 0.001; figure 2*a*). There was no significant effect of shadow presence (*t* = −1.66, *p* = 0.10) or an interaction between lighting type and shadow presence (*t* = 0.74, *p* = 0.46) on the proportion of time sea-slaters spent in the white background. For the full model, see electronic supplementary material, table S1.

Sea-slaters spent significantly more time in the grooves when the shadows were present (GzLM with quasibinomial error structure; *F*_1,75_ = 12.74, *p* < 0.001; figure 2*b*) irrespective of white or black background (*F*_1,75_ = 0.33, *p* = 0.57; figure 2*b*). For full and simplified models, see electronic supplementary material, tables S2 and S3.

### (b) Movement strategy

**Figure 3.**
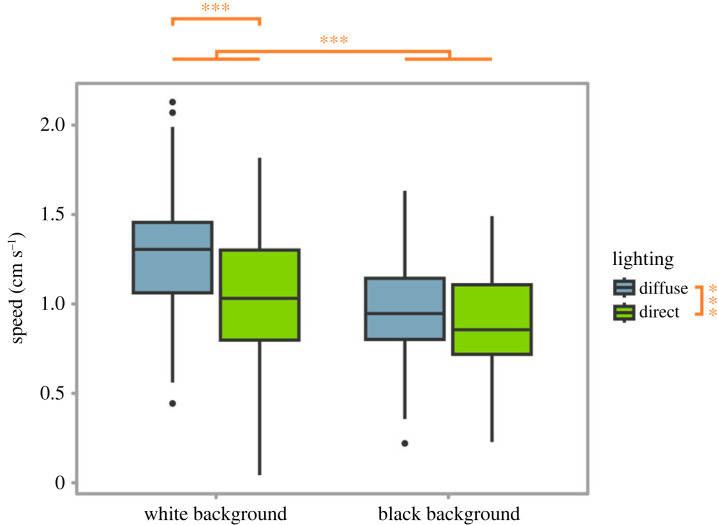
The speed of sea-slaters when located on different coloured backgrounds and under different types of lighting. Sea-slater speed was significantly greater on white backgrounds as opposed to black backgrounds and under diffuse lighting as opposed to direct lighting, with the difference between diffuse and direct lighting being greater on the white background. Boxes represent the median and interquartile range, and whiskers represent the range of the dataset. *** indicates *p* < 0.001.

Sea-slaters moved significantly faster under diffuse light than direct light (mixed effects model, *χ*^2^ = 11.27, d.f. = 1, *p* < 0.001; figure 3) and also moved faster on white than black backgrounds (*χ*^2^ = 118.66, d.f. = 1, *p* < 0.001; figure 3). The interaction between light and background colour was significant, diffuse light caused sea-slaters to move faster when on a white background, but not on a black background (*χ*^2^ = 28.96, d.f. = 1, *p* < 0.001; figure 3). There was no significant effect of a three-way interaction (*p* = 0.14), or any other two-way interactions involving shadow presence (*p* = 0.48, *p* = 0.19; for interactions between shadow presence and lighting type, and between shadow presence and background colour, respectively), or an effect of shadow presence itself (*p* = 0.71) on sea-slater speed. For full and simplified models, see electronic supplementary material, tables S4 and S5.

**Figure 4.**
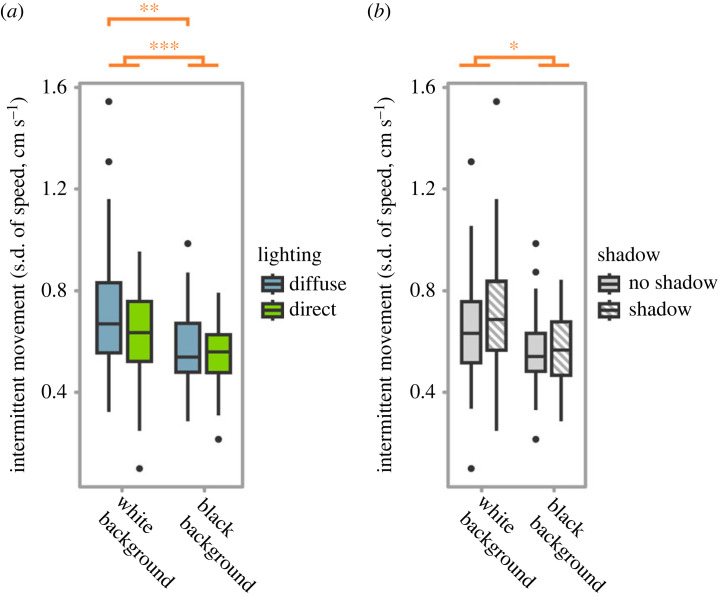
(*a*) Intermittent movement of sea-slaters when located on different coloured backgrounds and under different types of lighting. Movement was significantly more intermittent on white backgrounds as opposed to black backgrounds. There was also an interaction between lighting and background colour, with movement being significantly more intermittent under diffuse lighting as opposed to direct lighting on the white background. (*b*) Intermittent movement in the presence or absence of shadows. Movement was significantly more intermittent when shadows were present, with this effect being stronger when sea-slaters were located on the white background as opposed to the black background. Boxes represent the median and interquartile range, and whiskers represent the range of the dataset.*, ** and *** indicate *p* < 0.05, < 0.01 and less than 0.001, respectively.

Sea-slater movement was more intermittent (s.d. of speed) on white than black backgrounds (mixed effects model, *χ*^2^ = 114.42, d.f. = 1, *p* < 0.001; figure 4*a*). Lighting interacted with background colour, with sea-slater movement being significantly more intermittent under diffuse light on white backgrounds compared to diffuse light on black backgrounds (*χ*^2^ = 9.71, d.f. = 1, *p* = 0.002; figure 4*a*). Shadow presence resulted in significantly more intermittent motion, and this effect was stronger when sea-slaters were located on the white rather than the black background (*χ*^2^ = 4.96, d.f. = 1, *p* = 0.03; figure 4*b*). There was no significant effect of a three-way interaction (*p* = 0.33) or a two-way interaction between lighting type and shadow presence (*p* = 0.40). For full and simplified models, see electronic supplementary material, tables S6 and S7.

### (c) Colour change

**Figure 5.**
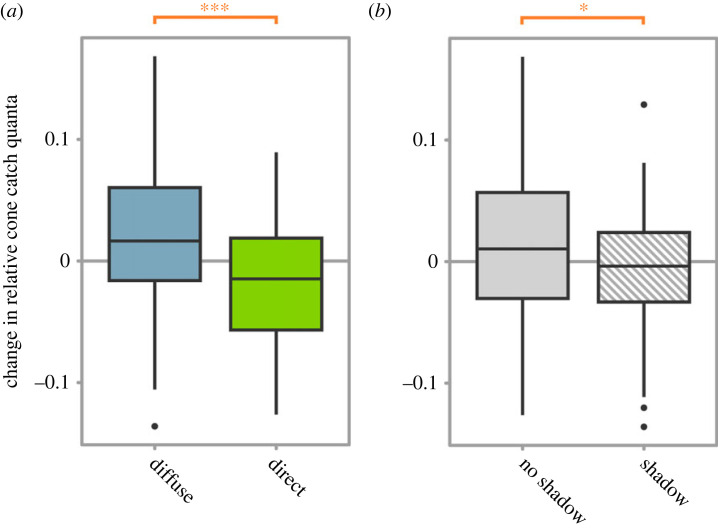
(*a*) The change in luminance of sea-slaters under diffuse versus direct lighting. Sea-slaters became significantly lighter under diffuse lighting and significantly darker under direct lighting. (*b*) The change in luminance of sea-slaters in the presence versus absence of shadows. Sea-slaters became significantly lighter when shadows were absent and significantly darker when shadows were present. Boxes represent the median and interquartile range, and whiskers represent the range of the dataset.* and *** indicate *p* < 0.05 and less than 0.001, respectively.

Sea-slaters became lighter coloured under diffuse lighting and darker under direct lighting, with a highly significant effect (GLM, *F*_1,158_ = 38.09, *p* < 0.001; figure 5*a*). Sea-slaters also became significantly lighter coloured when shadows were absent compared to when shadows were present, although this effect was comparatively weak (*F*_1,158_ = 4.15, *p* = 0.04; figure 5*b*).

**Figure 6.**
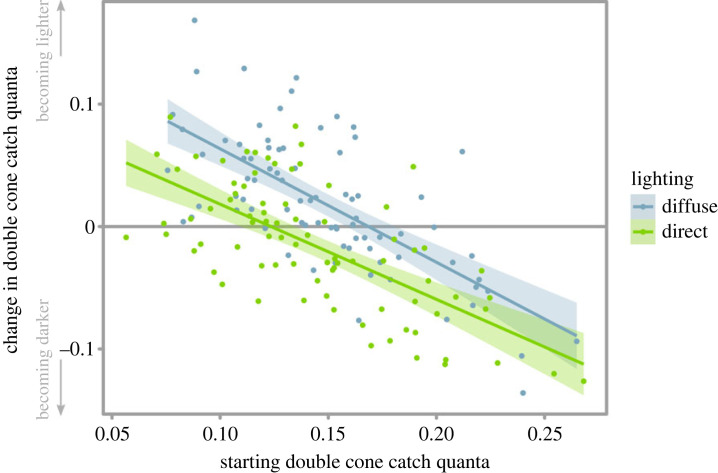
The change in luminance of sea-slaters relative to their starting luminance, under both direct and diffuse lighting types. While there was a significant negative correlation between the change in luminance of sea-slaters and their starting luminance, this rate of change was not significantly different for each type of lighting. Lines represent least-squares linear regression models and shaded areas represent 95% confidence intervals.

There was also a significant negative correlation between the change in luminance and the starting luminance (*F*_1,158_ = 135.67, *p* < 0.001; figure 6), with sea-slaters changing luminance to be darker if they started off light, and changing luminance to be lighter if they started off dark. This model also shows that sea-slaters increased in luminance (became lighter coloured) under diffuse light as above (*p* = 0.003); however, the degree of change from starting luminance (i.e. rate of change) did not show an interaction with light type (*p* = 0.32; figure 6). There was also no significant effect of any three-way (*p* = 0.25) or two-way interactions (*p* = 0.54 and *p* = 0.23; on interactions between shadow presence and starting luminance, and shadow presence and lighting type respectively) on the luminance change of sea-slaters. For full and simplified models, see electronic supplementary material, tables S8 and S9.

## 4. Discussion

The structure of the light environment caused both morphological and behavioural anti-predator responses in the marine isopod *Ligia oceanica*, together with interactions from substrate colour and 3D habitat shape. Under diffuse lighting, sea-slaters exhibited more risk-averse behaviours than under direct lighting, preferring the black background, moving faster, and having more pronounced intermittent movement. These movement responses were especially exaggerated when sea-slaters were present on the white background, which was on average a worse luminance match to their body colour. Despite this, diffuse light caused the sea-slaters to change colour to become lighter. The sea-slaters also appeared to make use of the shaded microhabitats when they were present, with individuals spending more time in the shadows and turning a darker colour when shadows were present (shadow presence was a function of both lighting and 3D habitat shape in our experiment).

These risk-averse behavioural responses align with our predictions based on the ecology of this species and its common predators, and match the typical anti-predator responses in other species [58]. The behaviours are likely to help protect prey from predators such as shorebirds and seabirds that increase their visually guided searching under higher nocturnal light levels, whether artificial [17,18,60,62] or natural [64,65]. This may reduce mortality from predation (e.g. as has been demonstrated in stream ecosystems where invertebrates avoid drifting in the main water column under ALAN [66]); however, these behaviours are ultimately likely to reduce fitness by restricting the sea-slaters' own foraging rates or limiting accessible habitat. Assessing how the behaviours observed in our experimental chambers translate into real-world habitat use is also difficult. While our treatment effects were highly statistically significant, the absolute effect sizes were comparatively small. This will primarily reflect the sea-slater's constant search behaviour in the experimental chambers, rarely stopping to shelter and therefore covering considerable ground during each trial.

A key finding of our study is that diffuse lighting causes exaggerated responses in the sea-slaters. This may be because bright, diffuse lighting is rarely found naturally at night; it will only occur with a bright moon and very thin cloud cover or fog, so may not have exerted significant selective pressure [67]. Comparatively intense diffuse lighting is much more likely to exist as a product of ALAN, whether through multiple distributed sources or skyglow [2,49,68,69]. Numerous studies suggest lunar cues are masked to a great enough extent that it can severely disrupt a range of behavioural and morphological processes in a variety of species [7,10,70]. Thus, the exaggerated reactions of sea-slaters to diffuse lighting, along with the disconnect in sea-slaters' behavioural and morphological responses, could reflect the lack of selection pressure under these novel light environment structures.

While the behavioural responses of sea-slaters matched our adaptive hypothesis, their morphological colour change was directly counter to our predictions; diffuse lighting caused sea-slaters to become lighter even though they were a closer match to the black background, and they chose to spend more time on the black background. This disconnect between morphological and behavioural defence mechanisms appears maladaptive as a camouflage response [38,57], probably leaving them vulnerable to predation as luminance matching is fundamental to camouflage [71,72]. Evidence from treefrogs, flatfishes and newts shows that a mismatch between an individual's colour change and microhabitat choice dramatically increases its predation risk [38,39,73,74], implying diffuse light could increase sea-slater mortality.

This maladaptive colour change may be due to low-level sensory limitations or biases in the mechanisms used to infer substrate brightness. The colour of a surface or object can only be estimated by comparing the intensity of light illuminating the surface (irradiance) to the intensity of light being reflected. Isopods have been shown to use upwards- and downwards-facing photoreceptors in their eyes to estimate surface colour (comparing irradiance to reflectance) and trigger colour change [75–77]. We found that sea-slaters became lighter under diffuse light, implying they were either under-estimating the intensity of a diffuse illuminant, or over-estimating the reflectance of the shadowless substrate, or both. Future work could investigate their colour-matching system further through behavioural experiments controlling for contrast and light intensity, or neurophysiological investigations of their sensory systems.

Alternatively, our findings may be consistent with diffuse lighting being used as a cue for dawn, therefore disrupting the sea-slaters' circadian rhythm. Under natural conditions, they become lighter at dawn, which is thought to help regulate their hygrothermal balance [57,75]. ALAN has been shown to interfere with circadian rhythms in isopods [76] and other species [68]. Indeed, diffuse skyglow can reduce the night-time release of the hormone melatonin in a freshwater fish [78]. In isopods, this hormone triggers melanin dispersal in chromatophores (making the body darker) and regulates melanin production [76], so if the diffuse lighting disrupts its release in this way it will lead to the lighter body colour that we see as a result. However, our results do not fully support this hypothesis because sea-slaters were able to adjust their body colour in relative terms equally well under either diffuse or direct light (i.e. lack of interaction between rate of colour change and light treatment, figure 6), whereas a release of melatonin might be expected to reduce the response range of the chromatophores under diffuse light. Further work could investigate the time-of-day interference hypothesis by testing whether diffuse light also causes other dawn-like behaviour, or measuring melatonin levels directly.

A final explanation for the maladaptive colour change may be due to a risk-threshold being exceeded under diffuse lighting. ALAN is thought to influence the landscape of fear in birds [15] and affects gene expression and hormone releases seen in a wide range of species [79]. For example, dogwhelks *Nucella lapillus* experience increased metabolic rates under ALAN that lowers their survival [18], and rodents suffer impaired reproductive, cognitive and social abilities under ALAN [80,81]. While few studies have examined colour change responses to human disturbance, there is evidence in species such as shore crabs (*Carcinus maenas*) that certain anthropogenic interference, for example ship noise, prevents individuals changing colour to match their background [82]. This is likely because colour change is energetically costly [30], so if individuals are stressed they may preferentially divert limited energy reserves to more immediately important processes, such as movement and metabolism [83]. In sea-slaters this may lead to a ‘default’ change to be a lighter colour, as generally it is more energetically costly to disperse the pigments that make their body colour darker [73,76], while they otherwise seek out shelter. As above, this would likely affect the rate of colour change between treatments that we did not observe.

In conclusion, our study highlights the need to consider not only the intensity and spectrum of light emissions, but also its directional/structural properties in work investigating the impact of ALAN on behaviour and ecosystems. This implies future work should first seek to improve methodologies for quantifying light structure at night and, second, perform behavioural and morphological investigations that either simulate specific light structures, or use real-world variance. Finally, the long-term trophic effects should be examined in a wider ecological context. There have been a variety of methods proposed to reduce both skyglow specifically and ALAN more generally over recent years, including avoiding introduction of ALAN into new areas, restoring natural darkness in areas that have previously been artificially lit, minimizing ALAN wherever possible (e.g. through dimming, part-night lighting, improved shielding), and potentially offsetting the introduction of ALAN into some areas by its removal from others [84]. Our work highlights the potential importance of exploring the effects of diffuse ALAN in other systems to determine how widespread these effects could be in nature.

## Data Availability

Data and R code are included as electronic supplementary material [85].
